# Comparison of RNA-Seq analysis data between tracheal mite-infested and uninfested Japanese honey bees (*Apis cerana japonica*)

**DOI:** 10.1186/s13104-023-06381-4

**Published:** 2023-06-26

**Authors:** Akihiko Suzuki, Masaki Kamakura, Takuya Shiramata, Shinji Nakaoka, Yoshiko Sakamoto

**Affiliations:** 1 National Institute for Environmental Studies, Tsukuba, Ibaraki 305-8506 Japan; 2 Department of Biotechnology, Faculty of Engineering, Toyama Prefectural University, Imizu, Toyama 939-0398 Japan; 3 Department of Advanced Transdisciplinary Science, Hokkaido University, Sapporo, Hokkaido 060-0810 Japan

**Keywords:** Japanese honey bee, Tracheal mite, *Acarapis woodi*, RNA-Seq

## Abstract

**Objective:**

The purpose of this data set is to investigate differences in RNA-Seq transcriptome profiles between *Acarapis woodi*-infested and uninfested Japanese honey bees (*Apis cerana japonica*). The data set is strengthened by data collected from different body parts (head, thorax, and abdomen). The data set will support future studies of molecular biological changes in mite-infested honey bees.

**Data description:**

We collected 5 mite-infested and 5 uninfested *A*. *cerana japonica* workers from each of 3 different colonies (designated as A, B, and C). Workers were dissected into 3 body sites (i.e., heads, thoraces, and abdomen), and 5 of each body site were pooled together for RNA extraction, generating a total of 18 RNA-Seq samples (2 infection status × 3 colonies × 3 body sites). FASTQ data files of each sample that were generated by a DNBSEQ-G400 sequencer with the 2 × 100 bp paired-end sequencing protocol are available in the DDBJ Sequence Read Archive under accession number DRA015087 (RUN: DRR415616–DRR415633, BioProject: PRJDB14726, BioSample: SAMD00554139–SAMD00554156, Experiment: DRX401183–DRX401200). The data set is a fine-scale analysis of gene expression in the mite-infested *A*. *cerana japonica* workers because 18 RNA-Seq samples are separated by 3 body sites.

## Objective

*Acarapis woodi*, a tracheal mite that infests honey bees, was first reported in the European honey bee (*Apis mellifera*) in England in the early 1990s [1] and has since spread around the world. The mite feeds on bee haemolymph in the tracheal tubes of adult bees [2]. Heavy infestations of the mite result in colony losses during winter due to damage to the tracheal system [3]. In recent years, mite infestations have ceased to be epidemic, even in places once heavily infested [4].

*Acarapis woodi* was first recorded in Japan in 2010 and has since spread rapidly in the Japanese honey bee (*Apis cerana japonica*) over a wide range of the country [5], resulting in overwinter mortality of colonies in *A*. *cerana japonica* [6]. *Apis cerana japonica* is an indigenous honey bee subspecies of *A*. *cerana* and plays a crucial role in maintaining ecosystems as a pollinator [7]. Therefore, infestation by *A*. *woodi* is of serious concern in relation to the bee population and associated biodiversity. To clarify the influences of infestation by *A*. *woodi* on *A*. *cerana japonica*, a recent study investigated the behavioral and morphological features associated with mite removal from *A*. *cerana japonica* [8], but changes in gene expression networks have not been investigated.

The purpose of the data set in this study is to reveal differences in RNA-Seq transcriptome profiles between *A*. *woodi*-infested and uninfested *A*. *cerana japonica*. The goal is to use it to identify molecular biological changes that occur in mite-infested bees. The data set will also provide useful information about the conservation of other honey bees.

## Data description

We collected 5 mite-infested and 5 uninfested *A*. *cerana japonica* workers from each of 3 different colonies (designated as A, B, and C). Workers were dissected into 3 body sites (i.e., heads, thoraces, and abdomen), and 5 samples of each dissected body site were pooled together for RNA extraction, generating a total of 18 RNA-Seq samples (2 infection status × 3 colonies × 3 body sites) (Table 1) [9–27]. Mite infestation was determined from the presence of mites in tracheal tubes. Dissection of each bee was conducted in liquid nitrogen, and then all samples were stored at − 80 °C until RNA extraction.

Total RNA was extracted from each sample using an RNeasy Mini Kit (Qiagen, Hilden, Germany), following the manufacturer’s instructions. The concentration and quality of the extracts were confirmed with a QuantiFluor RNA system (Promega, Madison, WI, USA), Quantus Fluorometer (Promega), 5200 Fragment Analyzer system (Agilent Technologies, Santa Clara, CA, USA), and Agilent HS RNA kit (Agilent Technologies). The libraries were prepared by using an MGIEasy RNA Directional Library Prep Set (MGI Tech, Shenzhen, Guangdong, China), quantified with a QuantiFluor dsDNA System (Promega), and quality-checked with a dsDNA 915 Reagent kit (Advanced Analytical Technologies, Orangeburg, NY, USA). DNA nanoballs were prepared from the libraries using a DNBSEQ-G400RS High-Throughput Sequencing Set (MGI Tech), following the manufacturer’s protocol, and then DNA nanoball libraries were sequenced on a DNBSEQ-G400 sequencer (MGI Tech) with the 2 × 100-bp paired-end sequencing protocol.

Sequencing generated a total of 175,407,768 (average ± SD, 19,489,752 ± 2,329,627) reads from the mite-infested samples and 174,561,408 (19,395,712 ± 1,682,127) reads from the uninfested samples. The FASTQ data were quality-checked in FastQC v. 0.11.9 software [28]. The final 18 data sets of demultiplexed raw FASTQ data were deposited in the DNA Data Bank of Japan (DDBJ) Sequence Read Archive under accession number DRA015087 (Run: DRR415616–DRR415633, BioSample: SAMD00554139-SAMD00554156, BioProject: PRJDB14726).

**Table 1:** Overview of data files/data sets

Label	Name of data file/data set	File type	Data repository and identifier (DOI or accession number)
Data set 1	RNA-Seq of AW-JA-head	.fastq	DDBJ Sequence Read Archive (https://identifiers.org/insdc.sra:DRR415616) [9]
Data set 2	RNA-Seq of AW-JA-thorax	.fastq	DDBJ Sequence Read Archive (https://identifiers.org/insdc.sra:DRR415617) [10]
Data set 3	RNA-Seq of AW-JA-abdomen	.fastq	DDBJ Sequence Read Archive (https://identifiers.org/insdc.sra:DRR415618) [11]
Data set 4	RNA-Seq of AW-JB-head	.fastq	DDBJ Sequence Read Archive (https://identifiers.org/insdc.sra:DRR415619) [12]
Data set 5	RNA-Seq of AW-JB-thorax	.fastq	DDBJ Sequence Read Archive (https://identifiers.org/insdc.sra:DRR415620) [13]
Data set 6	RNA-Seq of AW-JB-abdomen	.fastq	DDBJ Sequence Read Archive (https://identifiers.org/insdc.sra:DRR415621) [14]
Data set 7	RNA-Seq of AW-JC-head	.fastq	DDBJ Sequence Read Archive (https://identifiers.org/insdc.sra:DRR415622) [15]
Data set 8	RNA-Seq of AW-JC-thorax	.fastq	DDBJ Sequence Read Archive (https://identifiers.org/insdc.sra:DRR415623) [16]
Data set 9	RNA-Seq of AW-JC-abdomen	.fastq	DDBJ Sequence Read Archive (https://identifiers.org/insdc.sra:DRR415624) [17]
Data set 10	RNA-Seq of Cont-JA-head	.fastq	DDBJ Sequence Read Archive (https://identifiers.org/insdc.sra:DRR415625) [18]
Data set 11	RNA-Seq of Cont-JA-thorax	.fastq	DDBJ Sequence Read Archive (https://identifiers.org/insdc.sra:DRR415626) [19]
Data set 12	RNA-Seq of Cont-JA-abdomen	.fastq	DDBJ Sequence Read Archive (https://identifiers.org/insdc.sra:DRR415627) [20]
Data set 13	RNA-Seq of Cont-JB-head	.fastq	DDBJ Sequence Read Archive (https://identifiers.org/insdc.sra:DRR415628) [21]
Data set 14	RNA-Seq of Cont-JB-thorax	.fastq	DDBJ Sequence Read Archive (https://identifiers.org/insdc.sra:DRR415629) [22]
Data set 15	RNA-Seq of Cont-JB-abdomen	.fastq	DDBJ Sequence Read Archive (https://identifiers.org/insdc.sra:DRR415630) [23]
Data set 16	RNA-Seq of Cont-JC-head	.fastq	DDBJ Sequence Read Archive (https://identifiers.org/insdc.sra:DRR415631) [24]
Data set 17	RNA-Seq of Cont-JC-thorax	.fastq	DDBJ Sequence Read Archive (https://identifiers.org/insdc.sra:DRR415632) [25]
Data set 18	RNA-Seq of Cont-JC-abdomen	.fastq	DDBJ Sequence Read Archive (https://identifiers.org/insdc.sra:DRR415633) [26]
Data file 1	Sample preparation protocol	.pdf	Figshare, 10.6084/m9.figshare.21521178.v1 [27]

## Limitations

- Due to the small sample size, this data set provides only fundamental information about molecular biological reactions against mite infestation in *A*. *cerana japonica*. Additional investigation will be needed.
- We did not investigate the presence of other honey bee pathogens, such as viruses and microsporidians. Thus, it might be undeniable that such pathogens have influenced the result of this data set.
- This data set is based on different 3 colonies, whose genetic background was not investigated. Therefore, care should be taken in comparison with *A*. *cerana japonica* from other colonies.

## Data Availability

The data described in this Data Note can be freely and openly acquired from the DDBJ Sequence Read Archive under accession number DRA015087 (RUN: DRR415616–DRR415633, BioProject: PRJDB14726, BioSample: SAMD00554139–SAMD00554156, Experiment: DRX401183–DRX401200) [9–26]. See Table 1 for details and links to each data.

## Abbreviations

DDBJ: DNA Data Bank of Japan
